# Kikuchi-Fujimoto Disease: Diagnostic Dilemma and the Role of Immunohistochemistry

**DOI:** 10.4021/jocmr2009.09.1258

**Published:** 2009-10-16

**Authors:** Mehboob Hassan, Afzal Anees, Sufian Zaheer

**Affiliations:** a Department of Pathology and Department of Surgery, J N Medical College, Aligarh Muslim University, Aligarh, Uttar Pradesh, 202002, India

## Abstract

Kikuchi-Fujimoto (KD) disease is the rare differential diagnoses of chronic cervical lymphadenopathy of unknown etiology. The findings of histopathology may be overlapping, in such condition immunohistochemistry has a definite role to play. Since Tubercular lymphadenopathy is the commonest cause for chronic cervical lymphadenopathy in developing and tropical country like India. Occasionally it is misdiagnosed, ignoring the other rare condition like KD if immunohistochemistry is not taken into consideration to differentiate. As a result the morbidity increases and cost of antitubercular treatment (ATT) in wrong diagnosed case is enormous. We report a similar case of misdiagnoses, non responsive to ATT, finally diagnosed as a case of Kikuchi-Fujimoto disease, did well after recommended treatment.

**Keywords:**

Necrotizing lymphadenitis; Chronic cervical lymphadenitis; Immunohistochemistry

## Introduction

Kikuchi-Fujimoto disease was first described independently by Kikuchi [1] and Fujimoto et al [2], as a group of patients presented with lymphadenitis shows focal reticulum cell hyperplasia, nuclear debris and phagocytosis and another with cervical sub acute necrotizing lymphadenitis, respectively. Hence, the entity is known as Kikuchi-Fujimotos disease or simply Kikuchis disease (KD). The clinical and histological features suggest that this disorder represents a common pattern of response to a variety of etiologies [3]. Histopathology and immunohistochemistry are essential for the diagnosis. We report a case of a female suffering from chronic cervical lymphadenopathy, taking anti tubercular treatment (ATT) with no response. Immunohistochemistry (IHC) finally reversed the diagnoses and Kikuchi-Fujimoto disease was confirmed and treated successfully.

## Case Report

**Figure 1:**
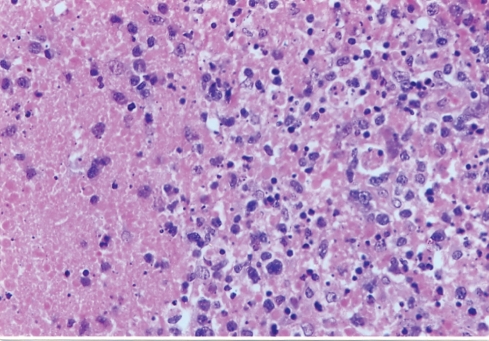
High power view of the lymph node demonstrating necrosis surrounded by karyorrhectic debris, histiocytes and plasmacytoid lymphocytes (H & E 40X).

**Figure 2:**
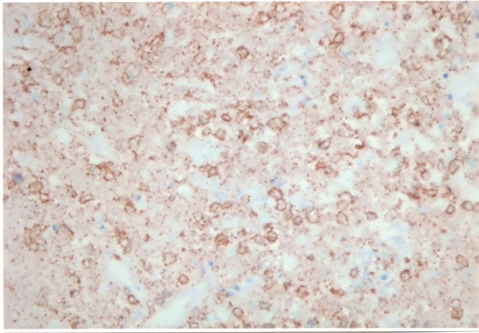
CD8 positvity of lymphocytic cells.

**Figure 3:**
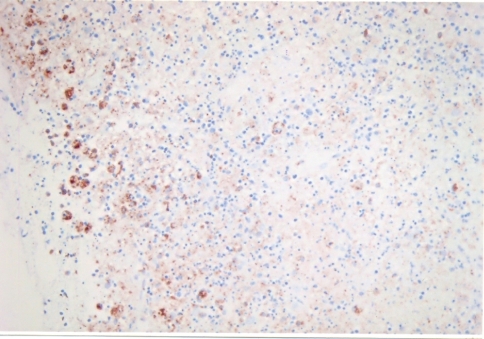
CD68 positvity of histiocytic cells.

A 27-year-old female was referred to our unit for 2 months history of swellings in the neck, fever with mild chills, night sweats, left axillary pain and weight loss of 4 5 kg. She was on ATT as advised by a local practitioner with no response for last 1 month. There was a family history suggestive of systemic lupus erythematosis (SLE). On physical examination, patient had pyrexia (temperature 38.8^o^C). Left cervical lymph nodes were significantly enlarged, mild tender, firm, and discrete. Left axillary lymph nodes were also enlarged. Other systemic examinations did not reveal any other abnormalities. Haematology showed mild pancytopaenia with normocytic red cell indices (Hb - 8.2 g/dl., MCV - 93.2 fl., Platelete count - 149 x 10^9^/l) and a relative lymphopaenia. Erythrocyte sedimentation rate (ESR) was 88 mm/h and C-reactive protein was 29. Serology for tuberculosis was negative. Excisional biopsy of a lymph node was done under local anesthesia. The histopathology demonstrated focal necrosis surrounded by karyorrhectic debris, histiocytes and plasmacytoid lymphocytes (Fig. 1). The subsequent Immunohistochemistry showed that the majority of cells in the affected foci were a mixture of CD8+ (Fig. 2) and CD68+ (Fig. 3) cells. Kikuchi-Fujimoto disease (KFD) was diagnosed. The patient showed the recovery after 21 days of supportive care.

## Discussion

Kikuchi-Fujimoto is also called histiocytic necrotizing lymphadenitis, often presents as a painful cervical lymphadenopathy in young females. Unilateral and posterior cervical lymph nodes are the commonest to be involved [4]. The course of the disease is variable, but usually self-limiting. Less common manifestations are in the form of axillary and mesenteric lymphadenopathy, splenomegaly, parotid gland enlargement, arthralgias, myalgias, aseptic meningitis [5], bone marrow haemophagocytosis [6] and interstitial lung disease [7]. The cutaneous lesions include erythematous macules, papules, plaques and nodules [8]. It is kept in the differential diagnoses of lymphadenopathy like infections including tubercular lymphadenitis, lymphoma, and other tumors. In Kikuchi disease, there is proliferation of T-cells, which, at some point, enter into the cycle of programmed cell death (apoptosis) [9]. Initial studies hinted at Yersinis enterocolitica and toxoplasma gondii, however the absence of associated bacterial infection as well as the resolution of the illness without the use of antibiotics refute this hypothesis [10]. Viral infection has also been suspected, including Epstein-Barr virus (EBV), herpes virus 6, parainfluenza virus and cytomegalovirus. Serological tests including antibodies to EBV and other viruses have proved noncontributory [10]. Widespread activation of T-cell is also seen in some inflammatory joint diseases. The strongest link is with SLE, although the exact nature of the association has not yet been established [8]. Since some patients of KD have latter developed SLE and since the necrotic lesion in the lymph node is similar, it has been suggested that necrotizing histiocytic lymphadenitis may be a 'forme firste' of SLE [11].

Patients with this KikuchiFujimoto disease may develop anemia, leucopenia, atypical lymphocytosis and raised ESR [12].

Fine-needle aspiration cytology (FNAC) can be used to make cytologic diagnosis. Characteristic cytologic findings in KD include crescentic histiocytes, plasmacytoid monocytes, and extracellular debris [13].

The histology of KD is distinctive. There are patchy, irregular paracortical areas of coagulative necrosis without a polymorphonuclear leucocyte infiltration. The necrotic areas show prominent karyorrhectic debris, immunoblasts, histiocytes with characteristics C-shaped nuclei, some of which contain cellular debris, and plasmacytoid T-cells/monocytes [10].

In general, immunohistochemistry shows a positive immunostaining by antibodies Mac 387, KP1 (CD 68) and Ki M1P. In addition, a variable number of T-cells immunostained by antibody MT1 (CD 43) or UCHL1 and (CD 45RO). CD8+ T cells were identified by antibody CD8/144 in all lesions. Immunohistochemistry clearly differentiate this KD from other chronic cervical lymphadenopathies. KD is self-limiting, the symptoms may spontaneously disappear in 1 - 6 months. Corticosteroids have shown good results [14]. The current evidence suggests the role of ciprofloxacin [15], chloroquin and hydroxychloroquin in this disease.

In conclusion, immunohistochemistry (IHC) has revolutionized the existing diagnostic protocol. It has further strengthened the histopathology in making the diagnosis more accurate in many clinical entities like sarcomas, carcinomas, and chronic inflammatory conditions. In our case, it helped in differentiating Kikuchi-Fujimotos disease from tubercular lymphadenopathy and guided to stop the non responsive antitubercular treatment. Patient responded well of supportive treatment and recovered in 3 weeks.

## References

[1] Kikuchi M (1972). Lymphadenitis showing focal reticulum cell hyperplasia with nuclear debris and phagocytes.. Acta Hematol Jpn.

[2] Fujimoto Y, Kozima Y, Yamaguchi K (1972). Cervical subacute necrotizing lymphadenitis. A new clinicopathological entity. Naika.

[3] Onciu M, Medeiros LJ (2003). Kikuchi-Fujimoto lymphadenitis. Adv Anat Pathol.

[4] Poulose V, Chiam P, Poh WT (2005). Kikuchi's disease: a Singapore case series. Singapore Med J.

[5] Sato Y, Kuno H, Oizumi K (1999). Histiocytic necrotizing lymphadenitis (Kikuchi's disease) with aseptic meningitis. J Neurol Sci.

[6] Mahadeva U, Allport T, Bain B, Chan WK (2000). Haemophagocytic syndrome and histiocytic necrotising lymphadenitis (Kikuchi's disease). J Clin Pathol.

[7] Sharma OP (2001). Unusual systemic disorders associated with interstitial lung disease. Curr Opin Pulm Med.

[8] Yasukawa K, Matsumura T, Sato-Matsumura KC, Takahashi T, Fujioka Y, Kobayashi H, Shimizu H (2001). Kikuchi's disease and the skin: case report and review of the literature. Br J Dermatol.

[9] Unger PD, Rappaport KM, Strauchen JA (1987). Necrotizing lymphadenitis (Kikuchi's disease). Report of four cases of an unusual pseudolymphomatous lesion and immunologic marker studies. Arch Pathol Lab Med.

[10] Schnitzer B, Jaffe ES (1995). Reactive lymphoid hyperplasia. Surgical pathology of lymph node and related organs 2nd edition.

[11] Katayama K, Sato Y, Shima N, Qiu JC, Ishida K, Mori S, Miyamura M (2002). Enhanced chemosensitivity after intermittent hypoxic exposure does not affect exercise ventilation at sea level. Eur J Appl Physiol.

[12] Kuo TT (1995). Kikuchi's disease (histiocytic necrotizing lymphadenitis). A clinicopathologic study of 79 cases with an analysis of histologic subtypes, immunohistology, and DNA ploidy. Am J Surg Pathol.

[13] Tsang WY, Chan JK (1994). Fine-needle aspiration cytologic diagnosis of Kikuchi's lymphadenitis. A report of 27 cases. Am J Clin Pathol.

[14] Martinez-Vazquez C, Hughes G, Bordon J, Alonso-Alonso J, Anibarro-Garcia A, Redondo-Martinez E, Touza-Rey F (1997). Histiocytic necrotizing lymphadenitis, Kikuchi-Fujimoto's disease, associated with systemic lupus erythemotosus. QJM.

[15] Mahajan VK, Sharma NL (2004). Kikuchi-Fujimoto disease: immediate remission with ciprofloxacin. Int J Dermatol.

